# Quantitative genetics of trauma induced mortality in *Drosophila melanogaster*

**DOI:** 10.1038/s41437-026-00828-7

**Published:** 2026-02-20

**Authors:** Gwanwoou Yun, Ronchen Liu, Nathaniel P. Sharp

**Affiliations:** 1 Department of Genetics, University of Wisconsin-Madison, Madison, WI USA; 2 Department of Molecular and Human Genetics, Baylor College of Medicine, Houston, TX USA

**Keywords:** Quantitative trait, Evolutionary biology

## Abstract

Traumatic brain injury is a major cause of chronic neurological impairment worldwide, and there is evidence that both genetic and environmental variation contribute to the likelihood of recovery. Using an insect model of traumatic brain injury, we examined variation in the risk of mortality using quantitative genetic approaches applied previously for life history traits in *Drosophila melanogaster*. We quantified additive genetic variance for mortality risk using a controlled breeding design and found levels of variation consistent with existing data on major fitness components. We did not detect inbreeding depression for mortality risk, suggesting that this trait is not strongly affected by recessive deleterious alleles. To explain the high level of standing genetic variation, we considered whether mortality risk depends on the metabolic resources available to an individual, also known as “condition”. We manipulated condition by inducing random mutations and by restricting calories during larval development. We found that reduced condition due to both random mutations and resource limitation significantly increased the risk of mortality following trauma. Among inbred lines, greater mortality risk was associated with lower viability, fecundity and longevity, consistent with an effect of genome-wide genetic quality. Our results suggest that further consideration of individual condition would be valuable for understanding and predicting variation in the outcomes of traumatic brain injury.

## Introduction

Traumatic brain injury (TBI) is a significant global cause of neurological disability and mortality (Roozenbeek et al. 2013; Dewan et al. 2019). There is evidence that the outcome of TBI depends on genetic variation among individuals, with a heritability of 26% (Kals et al. 2022). The identification of specific loci contributing to TBI outcomes would be valuable, but genome-wide association studies have revealed that this phenotype is complex and likely influenced by variation at many loci (Gomez et al. 2021; Kals et al. 2022; Merritt et al. 2024). *Drosophila melanogaster* has emerged as a model system for studying TBI, allowing for high-throughput experiments investigating the genetics of this disorder (Fesharaki-Zadeh and Datta 2024). Fly models have revealed modifiers of TBI outcomes including genetic background (Katzenberger et al. 2013) and age (Katzenberger et al. 2023), and physiological consequences of TBI such as tissue barrier dysfunction (Katzenberger et al. 2015), innate immunity (Katzenberger et al. 2013; Swanson et al. 2020), and sleep (Barekat et al. 2016; van Alphen et al. 2022). There is a need for further study of the origin and maintenance of genetic variation that influences TBI outcomes.

Our objective was to consider TBI-induced mortality in *D. melanogaster* from a quantitative genetic perspective, using the “high-impact trauma” (HIT) model developed by Katzenberger et al. (2013). The trauma induced using this approach likely affects multiple organs, but traumatic brain injuries are likely a major cause of the resulting mortality (Katzenberger et al. 2015). Specifically, we sought to quantify additive genetic variance for mortality rate following TBI, and to test the influence of recessive genetic variation, spontaneous mutations, and individual condition. If mortality rate is influenced by recessive deleterious alleles, we would expect the risk to differ between inbred and outbred flies. In a previous study, crosses between inbred lines with high and low mortality resulted in strains with intermediate mortality (Katzenberger et al. 2015), suggesting that the differences between the parental lines were driven mainly by additive genetic variation. We tested for inbreeding depression in HIT mortality by sampling from an outcrossing population, thereby incorporating a wider array of standing genetic variation.

The additive genetic variation for a polygenic trait will largely determine the response to selection (Falconer and Mackay 1996; Walsh and Lynch 2018). Previous studies have demonstrated significant variation in HIT mortality among inbred lines (Katzenberger et al. 2013, 2015, 2023), which would reflect overall genetic variation (additive and non-additive). In addition to testing for inbreeding depression as described above, we sought to quantify additive genetic variance for HIT mortality using a half-sibling breeding design (Lynch and Walsh 1998). This approach allowed us to quantify the narrow-sense heritability for the trait and compare standardized variance values among traits. We were particularly interested in whether the level of genetic variation for HIT mortality is similar to life history traits that have been studied in flies. Additionally, we examined the HIT mortality values that have been reported previously for lines from the Drosophila Genetic Reference Panel (DGRP; (Mackay et al. 2012), in relation to other traits that have been measured for the same lines, allowing us to assess correlations among traits.

As with any other quantitative trait, standing heritable variation in HIT mortality could be attributable, at least in part, to deleterious mutation-selection balance (Houle et al. 1994, 1996; Charlesworth and Hughes 1999; Zhang and Hill 2005; Charlesworth 2015), with directional selection favoring lower mortality. If many loci contribute to this trait, random mutagenesis would cause increased susceptibility to mortality. Alternatively, if this trait is influenced by relatively few loci, we would be less likely to detect a significant effect of mutagenesis. If the trait is actually under stabilizing selection favoring intermediate values (e.g., due to a strong trade-off with another fitness component), selection would favor reduced variance; we would then expect random mutations to increase the trait variance, with little effect on the trait mean (Keightley and Hill 1988). We tested the effect of random mutations by exposing male flies to a chemical mutagen and then measuring HIT mortality in their offspring.

One way to account for the high levels of genetic variance often observed in major fitness components like viability and fecundity (Houle 1992) is to posit that these traits reflect the ability of an individual to acquire resources, which in turn is depends on many loci (Houle 1991; Rowe and Houle 1996; Tomkins et al. 2004; Hooper and Bonduriansky 2022). The “condition” of an individual can be affected by its genetic quality, but also by the availability of resources in its environment, though these influences may not be entirely concordant (Clark et al. 2012; Bonduriansky et al. 2015; Hooper and Bonduriansky 2022). We tested for an effect of environmentally induced variation in condition by rearing flies in high- or low-quality larval environments and measuring their susceptibility to HIT-induced mortality as adults.

We find that HIT mortality shows high levels of additive genetic variation and condition-dependence. Correlations with other traits suggest that HIT mortality is influenced by many loci that also contribute to variation in other fitness components, such that variation in this trait is maintained by a balance between mutation and purifying selection.

## Materials and methods

### Fly strains and culturing

We conducted all experiments with a population of flies derived from the Canton-S wild-type genetic background, obtained from the Bloomington Drosophila Stock Center (RRID:BDSC_64349) and maintained as a large outbreeding cage population (approximately 2000–3000 individuals) with overlapping generations since 2019. We maintained flies at 25 °C, under a 12:12 light:dark cycle, and conducted crosses under CO_2_ anesthesia using flies that were 2–5 days old post eclosion, with females collected as virgins. Except where noted, we cultured flies on defined medium (14.3 g/L agar, 92.3 g/L white sugar, 46 g/L debittered yeast, 7.4 g/L potassium sodium tartrate, 0.93 g/L potassium phosphate, 0.46 g/L sodium chloride, 0.46 g/L calcium chloride, 0.46 g/L magnesium chloride, 0.46 g/L iron sulfate, 0.5% propionic acid) in standard vials seeded with 3–5 pellets of live yeast.

### High-impact trauma and mortality index

We subjected flies to high-impact trauma (HIT) following an approach described in detail previously (Katzenberger et al. 2013). For each replicate, we transferred a group of flies to an empty vial without anesthesia and restricted them to the bottom quarter of the vial with a cotton ball. We then attached the vial to the free end of a metal spring clamped to a wooden board, deflected the spring at a right angle and released it, causing the vial to contact a polyurethane pad at approximately 3 m/s (Katzenberger et al. 2013). For each replicate vial we applied this procedure three times, separated by 5 min of recovery time. We then transferred flies into vials with media, incubated them for 24 h, and then scored the number of dead flies in each vial. In addition to vials of flies subjected to trauma, we generated control vials where matched flies were transferred into empty vials for the same amount of time as the treatment flies but were not subjected to trauma. This “sham” treatment allowed us to control for any variation in mortality not caused by trauma. For each experimental unit (e.g., flies from the same family), we calculated the mortality index (MI) as the proportion of dead flies in the treatment vial minus the proportion of dead flies in the sham vial, multiplied by 100. Our experiments vary somewhat in the number of flies per treated vial, and combine males and females, but there is evidence that these factors do not influence mortality (Katzenberger et al. 2013). In our experiments, each vial contained an approximately equal number of males and females. Variation in mortality among experiments may be attributable to different application of the HIT device among experimenters, but we held this factor constant within each experiment.

### Genetic variance

We used a half-sibling breeding design to estimate genetic variance in mortality following high-impact trauma, using flies collected at random from the Canton-S population. We first created multiple “families” with one male (“sire”) and three virgin females (“dams”) each. Following one day of mating, we discarded the males and placed the females in individual oviposition vials. From each oviposition vial we collected adult offspring and subjected half to the HIT treatment and half to the sham treatment, with an average of 79 flies per MI measure (range 56–109). Some females did not produce offspring; excluding such cases, this experiment was ultimately comprised of 55 sires, 133 dams, and 10,496 offspring, tested in two roughly equal blocks (average 191 offspring per family; range 120–259). We calculated MI for the offspring of each dam and determined the among-sire component of variance by fitting a linear mixed model using the *R* package lme4 (Bates et al. 2015). In a half-sibling breeding design, the additive genetic variance is given by four times the among-sire component of variance, assuming epistasis is negligible (Lynch and Walsh 1998).

### Inbreeding depression

To test for an effect of inbreeding on mortality following high-impact trauma, we first collected flies at random from the Canton-S population and created vials with one male and one virgin female each. From each of these “families” we collected male and female offspring as virgins. We then generated outbred flies by crossing males from one family with females from a different family; we generated inbred flies by crossing males and females from the same family (full siblings). We were ultimately able to obtain MI data for 34 outbred and 34 inbred groups of flies, with an average of 49 flies per MI measure (range 38–50; 3331 flies in total). We tested for an effect of inbreeding on mean MI using a t-test and compared coefficients of variation using a randomization test with 10,000 replicates.

### Mutagenesis

To test the impact of random mutations on mortality following high-impact trauma, we first collected random males and virgin females from the Canton-S population. We placed groups of males in empty vials for two hours to induce starvation and then transferred them into bottles containing filter paper saturated with either sugar water (1 g/mL sucrose), or sugar water with 3 mM methyl methanesulfonate (MMS). MMS is an alkylating agent and mutagen, which is known to generate point mutations and aberrations in flies (Ashburner et al. 2005). The dose we selected has been shown to increase the frequency of sex-linked recessive lethal alleles by 70-fold (Nivard et al. 1992). After 24 h, we combined treated males with virgin females, with one male per vial. We expect offspring from these vials to be wild type when sired by control males, or to be heterozygous for induced mutations when sired by mutagenized males. We were ultimately able to obtain MI data for 25 control and 25 mutagenized groups of flies, with an average of 46 flies per MI measure (range 32–50; 2316 flies in total). We tested for an effect of mutagenesis on MI using a t-test.

### Larval diet quality

To test the impact of larval diet quality on mortality following HIT, we first placed oviposition plates in the Canton-S cage overnight. Following egg hatching, we picked L1 larvae and placed them in groups of 50 in vials of our standard media (described above; “high quality” diet), or in vials containing media with half of the standard amounts of sugar and yeast (“low quality” diet). We collected adults emerging in these vials and preserved some for body mass measurements (*N* = 51 flies per sex and diet treatment) and used others for MI measurements. To measure body mass, we dried flies at 70 °C for 24 h and weighed them individually to the nearest 0.01 mg. We tested for effects of larval diet on body mass using t-tests (Welch t-test in the case of males, where Levene’s test indicated unequal variances).

In this assay, flies were collected in mass across vials in each treatment, and so there was no pairing of HIT vials and sham vials. We ultimately obtained mortality data for 38 hit vials and 30 sham vials from the high-quality diet treatment, and for 44 HIT vials and 43 sham vials from the low-quality diet treatment, with an average of 29.8 flies per vial (range 28–30; 4624 flies in total). We calculated MI for each HIT vial by subtracting the average mortality across all sham vials from the corresponding diet treatment. We tested for an effect of diet quality on MI using a t-test. An alternative analysis of mortality in all HIT and sham replicates using a quasi-binomial generalized linear model produced the same conclusions.

### Trait correlations

The rate of mortality following HIT has previously been investigated for inbred strains from the DGRP (Katzenberger et al. 2015), where a variety of other traits have also been measured. To understand the relationship between mortality from trauma and underlying genetic quality, we obtained publicly available data on additional life history traits from these same lines. Specifically, we considered data on survival to adulthood (viability), using the average across three temperatures tested (Ellis et al. 2014), and data on lifespan and lifetime fecundity, using values corrected for body size and block (Durham et al. 2014). Of the 179 DGRP lines with MI data (chosen at random by Katzenberger et al. 2015), data on viability were available for 49 cases, and data on lifespan and fecundity were available for 169 cases. We calculated Pearson correlation coefficients for pairs of traits and tested for significance using bootstrapping with 10,000 replicates.

## Results

### High levels of genetic variance

**Fig. 1:**
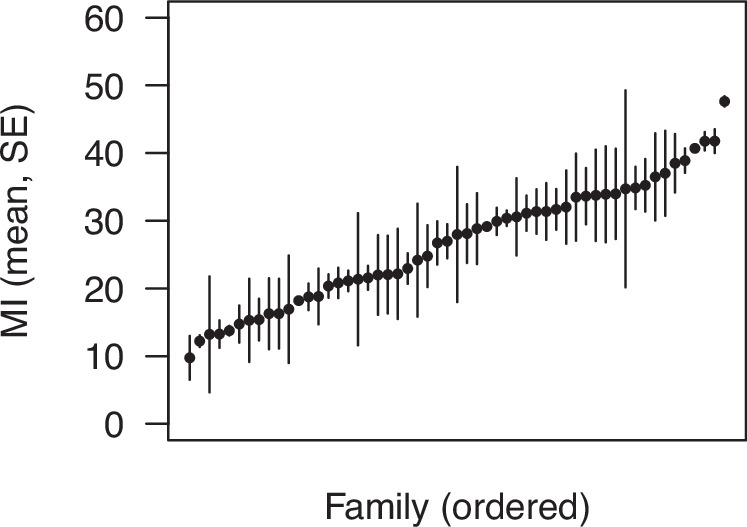
Genetic variance in mortality following high-impact trauma. Points represent means for 55 families of the mortality index (MI), ordered from low to high. Each family consisted of one male and 2–3 females, whose offspring were reared and tested separately. Error bars represent standard errors. We detected significant additive genetic variance in MI.

We detected significant additive genetic variance in HIT mortality in the Canton-S laboratory population (*χ*^2^ = 10.31, df = 1, *P* = 0.001; data in Table S1). We estimate a coefficient of additive genetic variation (Houle 1998) of 46.2% and a narrow sense heritability of 68.4%. The range of mean MI by sire we observed (Fig. 1) is consistent with the range observed for DGRP lines (Katzenberger et al. 2015). For comparison, we calculated the coefficient of total genetic variance for HIT mortality among the DGRP inbred lines studied by Katzenberger et al. (2015) and found a value of 36.9%. Thus, two studies of different fly populations using different methods both indicate relatively high levels of standing genetic variation for HIT mortality.

### No detectable inbreeding depression

**Fig. 2:**
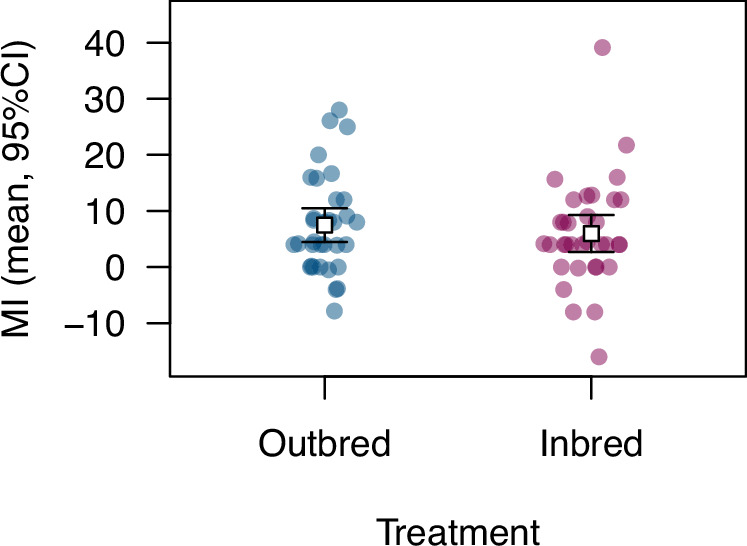
Effect of inbreeding on mortality following high-impact trauma. Inbred flies were the result of mating between full siblings; outbred families were the result of mating between unrelated individuals. Error bars represent 95% confidence intervals. We did not detect a significant effect of inbreeding on MI.

Inbreeding could affect mortality following HIT if there are partially recessive deleterious alleles contributing to variation in this trait, or if there is heterozygote advantage (Charlesworth and Willis 2009). Our analysis comparing MI in families derived from full-sibling mating (inbred) and outbred families (Fig. 2; data in Table S2) revealed no effect of inbreeding on the trait mean (*t* = 0.69, df = 66, *P* = 0.49; effect size 95% confidence interval: –2.87–5.89), although there was significant HIT-induced mortality overall (*t* = 6.17, df = 67, *P* = 4.56 × 10^–8^; mean MI 6.7%). If recessive alleles have both positive and negative effects on HIT mortality, we might expect greater trait variance among outbred families. The coefficient of variation in MI was greater in inbred families than in outbred families (158.4% versus 114.7%), but this difference was not statistically significant (randomization, *P* = 0.31). The observation that inbreeding did not significantly affect the mean or variance in HIT mortality suggests that most genetic variants affecting HIT mortality do so in an additive manner.

### Mutagenesis increases HIT mortality

**Fig. 3:**
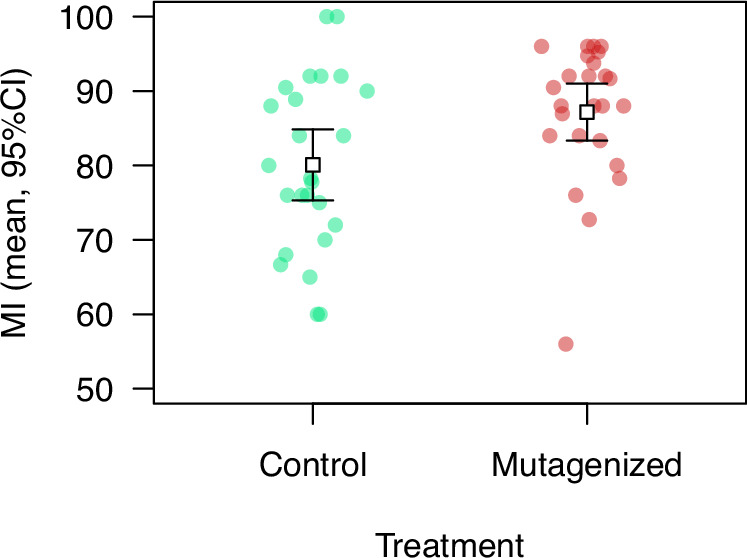
Effect of mutagenesis on mortality following high-impact trauma. Mutagenized families were derived from males exposed to starvation followed by sugar water containing the mutagen MMS; control families were derived from males exposed to starvation followed by sugar water only. Error bars represent 95% confidence intervals. Mutagenesis resulted in a significant increase in MI.

Standing genetic variation in mortality following HIT could be maintained in part by mutation-selection balance if this trait is a large “mutational target”. We found that the average MI was significantly higher (8.8%) in mutagenized families than in non-mutagenized families (Fig. 3; data in Table S3; *t* = –2.39, df = 48, *P* = 0.021). We did not detect an effect of mutagenesis on the coefficient of variation for MI (randomization with 10,000 replicates, *P* = 0.19), though existing genetic variation could have partially obscured a signal of increased variation following mutagenesis. The dose of MMS we used, which has been found to have a relatively weak effect on heterozygous viability (Melde et al. 2024), was nevertheless sufficient to significantly affect HIT mortality, suggesting that many loci contribute to the outcome of TBI.

### Low quality larval diet increases HIT mortality

**Fig. 4:**
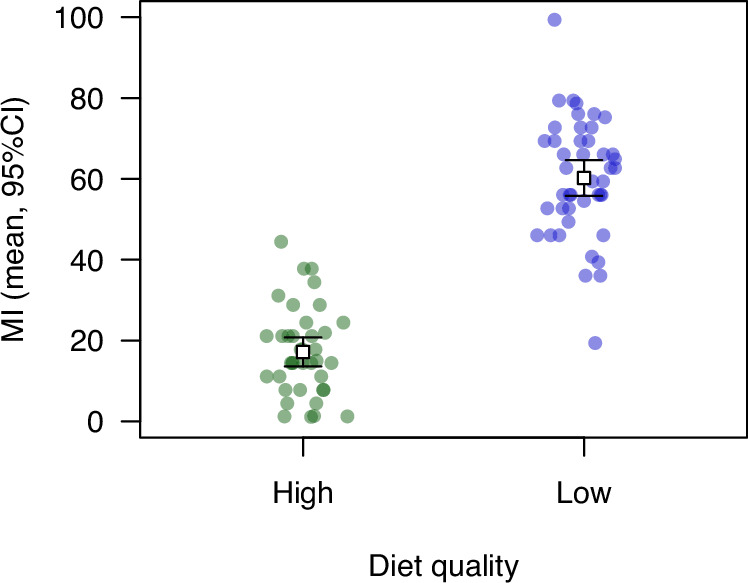
Effect of larval diet quality on mortality following high-impact trauma. Flies reared from the L1 larval stage on a low-quality diet (half of the standard amount of sugar and yeast) displayed higher MI than flies reared on a standard diet. Error bars represent 95% confidence intervals.

Just as random mutations could influence HIT mortality by affecting the ability of individuals to acquire resources, development in a resource-limited environment could also influence the outcomes of trauma. We reared flies on either standard media or media with half of the usual amount of yeast and sugar; we found that the low-quality diet reduced female body mass by 20% (data in Table S4; *t* = 8.09, df = 100, *P* = 1.48 × 10^–12^) and reduced male body mass by 16% (data in Table S4; Welch *t* = 8.25, df = 83.23, *P* = 2.00 × 10^–12^). All else being equal, we would expect a reduction in body mass to lead to a lower rate of HIT mortality, since smaller flies experience less force during the HIT procedure (Johnson-Schlitz et al. 2022). However, we found that average MI was 3.5-fold higher in flies reared on the low-quality larval diet (Fig. 4; data in Table S5; *t* = 14.9, df = 80, *P* < 2.2 × 10^–16^), indicating that HIT mortality is highly sensitive to variation in resource availability during larval development, counteracting any direct influence of body size.

### HIT mortality is correlated with fitness components

**Fig. 5:**
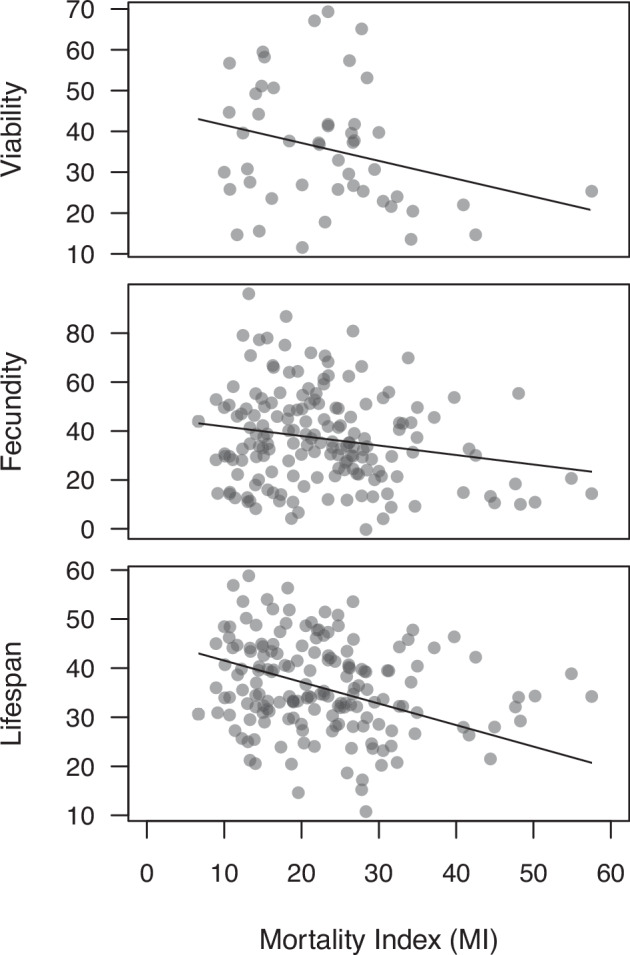
Relationships between fitness-related traits and mortality following high-impact trauma. Analysis of published data on DGRP lines (Durham et al. 2014; Ellis et al. 2014; Katzenberger et al. 2015) revealed significant negative correlations between MI and viability (top), fecundity (middle) and lifespan (bottom). Linear model trendlines are shown in each panel. Viability represents the average percentage of eggs that developed into adults across three temperatures (see Ellis et al. 2014 for details). Fecundity represents total eggs laid in two-day periods in weeks 1, 3, 5, and 7 of adulthood, corrected for body size and block (see Durham et al. 2014 for details). Lifespan represents the number of days of female survival (see Durham et al. 2014 for details).

Our findings above suggest that mortality following HIT is affected by random mutations and condition, which are underlying variables that are also expected to affect fitness related traits. We therefore predicted that HIT mortality would be negatively correlated with fitness-related traits across genotypes. Based on analyses of sets of DGRP lines where both MI and fitness information is available, we confirmed this prediction (Fig. 5). Specifically, we found that MI was negatively correlated with egg-to-adult viability (*r* = –0.28, bootstrap *P* = 0.029, bootstrap 95% CI: –0.50 to –0.02), lifetime fecundity (*r* = –0.20, bootstrap *P* = 0.010, bootstrap 95% CI: –0.33 to –0.05), and lifespan (*r* = –0.20, bootstrap *P* = 0.002, bootstrap 95% CI: –0.32 to –0.08). The mortality risk in HIT-treated flies is therefore greater in genotypes that have lower fitness in the absence of HIT.

Previous investigations of the relationship between MI and genotype in the DGRP lines revealed a common genetic variant in the gene *grh* which was associated with elevated mortality following HIT (Katzenberger et al. 2015). To determine whether the above trait relationships persist in the absence of this variant, we repeated our analyses excluding 11 DGRP lines harboring *grh* variants and obtained similar results for viability (*r* = –0.28, bootstrap *P* = 0.056), fecundity (*r* = –0.21, bootstrap *P* = 0.008) and lifespan (*r* = –0.24, bootstrap *P* = 2 × 10^–4^). Variation at the *grh* gene therefore does not account for the overall genetic correlation between HIT mortality and fitness-related traits.

## Discussion

We used an established insect model to investigate the quantitative genetics of mortality following traumatic injury. There appears to be substantial additive genetic variation for this trait (Fig. 1), but we did not find evidence for inbreeding depression (Fig. 2); considering the coefficient of additive genetic variation, which uses the trait mean for standardization, genetic variation in HIT mortality (46.2%) is within the range of previous reports on *Drosophila* life history traits like male mating success, female fecundity, and larval viability (Houle 1992, 1998; Charlesworth and Hughes 1999; Sharp and Agrawal 2018). This suggests that variation in HIT mortality has a broad genetic basis, as with major fitness components.

We should consider factors in our experiment that could cause genetic variance in HIT mortality to be over- or under-estimated. First, we measured genetic variance in a population derived from Canton-S, which has likely experienced population bottlenecks during its history as a lab strain. While our lab population is unlikely to be in mutation-selection equilibrium, it has been comprised of thousands of individuals for several years, allowing genetic variation to accumulate. Nevertheless, we might expect reduced standing genetic variation relative to wild populations (but see below for comparisons with DGRP data). Second, while the among-sire variance in a half-sib breeding design largely reflects additive genetic variation, additive-by-additive epistatic variation can also contribute to a smaller extent (Falconer and Mackay 1996; Lynch and Walsh 1998). A high degree of epistatic variance for HIT mortality (i.e., due to strong interactions between common alleles) could therefore upwardly bias our estimate of additive genetic variance (Hill et al. 2008). In any case, our results do not preclude the existence of alleles with epistatic or dominance effects on HIT mortality but rather suggest that alleles with additive effects account for much of the phenotypic variation in this trait. Finally, the evidence for condition-dependence suggests that variation in larval density among oviposition vials could contribute to total variation in HIT mortality, but offspring totals among dams conformed to a Poisson distribution (Kolmogorov-Smirnov test: *D* = 0.09, *P* = 0.20), and we did not detect genetic variation for the total offspring (*χ*^2^ = 0, df = 1, *P* = 1), indicating that density variation was minimal and did not contribute to our estimate of genetic variance.

Our test for inbreeding depression would not be effective if alleles affecting HIT mortality are disproportionately likely to be recessive lethal, since such alleles would be purged prior to our testing of adult flies. Previous studies indicate that recessive lethality cannot be the sole cause of inbreeding depression for life history traits in *Drosophila* (Latter et al. 1995; Fry et al. 1998; Charlesworth et al. 2007; Mallet and Chippindale 2011). It is unclear whether HIT mortality is an exception to this pattern. We may have had low power to detect inbreeding depression with *n* = 34 per group; simulations using the variance in MI (regardless of treatment) observed in this part of our study indicate that a future experiment with this sample size would have <50% power to detect an MI increase under inbreeding of 60% or less. We also observed lower overall mortality here (Fig. 2) than in other parts of our study, though it was still significant. We therefore cannot rule out the presence of mild or moderate inbreeding depression for HIT mortality, which might be detectable using larger samples, higher levels of trauma, or a greater coefficient of relatedness.

Laboratory populations may not always be representative of natural populations, and so we compared our genetic variance estimate with data from Katzenberger et al. (2015), who characterized variance among inbred DGRP lines. If there is little dominance variance for this trait in the DGRP population, then the variance among DGRP lines should mainly reflect additive genetic variation (or additive-by-additive epistasis). We calculated a standardized genetic variance among DGRP lines of 36.9%, similar to what we observed. Lower genetic variance in the DGRP lines would be expected if HIT mortality is affected by recessive deleterious alleles that were purged during inbreeding; crosses between DGRP lines with very different trait values resulted in progeny with trait values near the midparent value (Katzenberger et al. 2015), suggesting a lack of directional dominance effects following inbreeding. We conclude that there is a relatively high level of genetic variation for mortality following traumatic injury in flies, in both laboratory and wild populations, and that much of this variance is likely additive.

If HIT mortality acts as a condition-dependent trait, the risk of mortality could increase following random mutagenesis or diet restriction. We found that flies that inherited mutagenized chromosomes were significantly more susceptible to mortality following HIT (Fig. 3), suggesting that this trait is a large mutational target (Houle et al. 1996; Houle 1998) and that natural selection has historically favored alleles whose effects include reduced HIT mortality. In our study, the flies tested would carry induced mutations in the heterozygous state, with the exception of any Y-linked mutations; although we did not detect inbreeding depression involving standing variance, some induced mutations could still have recessive effects on HIT mortality that would be revealed in homozygous flies.

We also found that flies reared on a low-quality larval diet were substantially more susceptible to mortality following HIT (Fig. 4). Our diet manipulation reduced adult dry body mass by 16–20%; for comparison, a diet manipulation causing a similar body mass reduction detectably reduced male sperm competition success (Clark et al. 2012). The increase in HIT mortality in the presence of both random mutations and reduced larval diet quality suggests that HIT mortality is condition dependent. This is concordant with results from rodent models, which indicate that negative TBI outcomes are exacerbated by early life stress (Fesharaki-Zadeh et al. 2020; Sanchez et al. 2021). While we chose to test mixed-sex groups of flies, given previous evidence that sex does not play a significant role in HIT mortality, (Katzenberger et al. 2013), males and females differ in body size and could respond differently to environmental and genetic stressors, so further investigation of sex-specific HIT mortality would be valuable.

If HIT mortality depends on genetic quality, we would predict that genotypes that are more susceptible to HIT would also show reduced values of key life history components. We were able to confirm this prediction by examining trait correlations for sets of DGRP lines: higher HIT mortality was associated with lower viability, fecundity, and lifespan (Fig. 5). There is also evidence that older flies are more susceptible to HIT (Katzenberger et al. 2013), consistent with the increased expression of deleterious alleles with age predicted under the mutation accumulation hypothesis for the evolution of aging (Charlesworth 2000; Li et al. 2023).

Taken together, our findings indicate that flies with reduced condition––either because of deleterious genetic variants or because of reduced access to resources during development––are at increased risk of mortality following traumatic injury. In humans, there is growing interest in the complex ways that social, demographic and environmental factors may influence recovery from TBI (Ponsford 2013; Haarbauer-Krupa et al. 2021). We suggest that further consideration of individual condition alongside specific risk factors may be a useful framework for explaining and predicting variation in TBI outcomes.

## Supplementary information

Table S1

Table S2

Table S3

Table S4

Table S5

## Data Availability

The data associated with this study are available as Supplementary Material.

## References

[CR1] van Alphen B, Stewart S, Iwanaszko M, Xu F, Li K, Rozenfeld S et al. (2022) Glial immune-related pathways mediate effects of closed head traumatic brain injury on behavior and lethality in Drosophila. PLoS Biol 20:e3001456. 35081110 10.1371/journal.pbio.3001456 PMC8791498

[CR2] Ashburner M, Golic KG, Hawley RS (2005) Drosophila: a laboratory handbook. 2nd edn. Cold Spring Harbor Laboratory Press, Cold Spring Harbor, NY.

[CR3] Barekat A, Gonzalez A, Mauntz RE, Kotzebue RW, Molina B, El-Mecharrafie N et al. (2016) Using Drosophila as an integrated model to study mild repetitive traumatic brain injury. Sci Rep 6:25252. 27143646 10.1038/srep25252 PMC4855207

[CR4] Bates D, Mächler M, Bolker B, Walker S (2015) Fitting linear mixed-effects models using lme4. J Stat Softw 67:1–48.

[CR5] Bonduriansky R, Mallet MA, Arbuthnott D, Pawlowsky-Glahn V, Egozcue JJ, Rundle HD (2015) Differential effects of genetic vs. environmental quality in Drosophila melanogaster suggest multiple forms of condition dependence. Ecol Lett 18:317–326. 25649176 10.1111/ele.12412

[CR6] Charlesworth B (2000) Fisher, Medawar, Hamilton and the evolution of aging. Genetics 156:927–931. 11063673 10.1093/genetics/156.3.927 PMC1461325

[CR7] Charlesworth B (2015) Causes of natural variation in fitness: evidence from studies of Drosophila populations. Proc Natl Acad Sci USA 112:1662–1669. 25572964 10.1073/pnas.1423275112 PMC4330759

[CR8] Charlesworth B, Hughes KA (1999) The maintenance of genetic variation in life-history traits. In: Singh R, Krimbas C (eds) Evolutionary genetics: From molecules to morphology, Cambridge University Press: Cambridge UK.

[CR9] Charlesworth B, Miyo T, Borthwick H (2007) Selection responses of means and inbreeding depression for female fecundity in Drosophila melanogaster suggest contributions from intermediate-frequency alleles to quantitative trait variation. Genet Res 89:85–91. 17521472 10.1017/S001667230700866X

[CR10] Charlesworth D, Willis JH (2009) The genetics of inbreeding depression. Nat Rev Genet 10:783–796. 19834483 10.1038/nrg2664

[CR11] Clark SCA, Sharp NP, Rowe L, Agrawal AF (2012) Relative effectiveness of mating success and sperm competition at eliminating deleterious mutations in drosophila melanogaster. Plos ONE 7:e37351. 22662148 10.1371/journal.pone.0037351 PMC3360693

[CR12] Dewan MC, Rattani A, Gupta S, Baticulon RE, Hung Y-C, Punchak M et al. (2019) Estimating the global incidence of traumatic brain injury. J Neurosurg 130:1080–1097. 29701556 10.3171/2017.10.JNS17352

[CR13] Durham MF, Magwire MM, Stone EA, Leips J (2014) Genome-wide analysis in Drosophila reveals age-specific effects of SNPs on fitness traits. Nat Commun 5: 4338. 25000897 10.1038/ncomms5338

[CR14] Ellis LL, Huang W, Quinn AM, Ahuja A, Alfrejd B, Gomez FE et al. (2014) Intrapopulation genome size variation in D. Melanogaster reflects life history variation and plasticity. PLoS Genet 10:e1004522. 25057905 10.1371/journal.pgen.1004522 PMC4109859

[CR15] Falconer DS, Mackay TFC (1996) Introduction to quantitative genetics. 4th edn. Longman Group Ltd, Essex UK.

[CR16] Fesharaki-Zadeh A, Datta D (2024) An overview of preclinical models of traumatic brain injury (TBI): relevance to pathophysiological mechanisms. Front Cell Neurosci 18:1371213. 38682091 10.3389/fncel.2024.1371213 PMC11045909

[CR17] Fesharaki-Zadeh A, Miyauchi JT, St Laurent-Arriot K, Tsirka SE, Bergold PJ (2020) Increased behavioral deficits and inflammation in a mouse model of co-morbid traumatic brain injury and post-traumatic stress disorder. ASN Neuro 12:1759091420979567. 33342261 10.1177/1759091420979567 PMC7755938

[CR18] Fry JD, Heinsohn SL, Mackay TFC (1998) Heterosis for viability, fecundity, and male fertility in drosophila melanogaster: comparison of mutational and standing variation. Genetics 148:1171–1188. 9539433 10.1093/genetics/148.3.1171 PMC1460047

[CR19] Gomez A, Batson C, Froese L, Zeiler FA (2021) Genetic variation and impact on outcome in traumatic brain injury: an overview of recent discoveries. Curr Neurol Neurosci Rep 21:19. 33694085 10.1007/s11910-021-01106-1

[CR20] Haarbauer-Krupa J, Pugh MJ, Prager EM, Harmon N, Wolfe J, Yaffe K (2021) Epidemiology of chronic effects of traumatic brain injury. J Neurotrauma 38:3235–3247. 33947273 10.1089/neu.2021.0062 PMC9122127

[CR21] Hill WG, Goddard ME, Visscher PM (2008) Data and theory point to mainly additive genetic variance for complex traits. PLoS Genet 4:e1000008. 18454194 10.1371/journal.pgen.1000008 PMC2265475

[CR22] Hooper AK, Bonduriansky R (2022) Effects of genetic vs. environmental quality on condition-dependent morphological and life history traits in a neriid fly. J Evol Biol 35:803–816. 35514040 10.1111/jeb.14014 PMC9325454

[CR23] Houle D (1991) Genetic covariance of fitness correlates: what genetic correlations are made of and why it matters. Evolution 45:630. 28568816 10.1111/j.1558-5646.1991.tb04334.x

[CR24] Houle D (1992) Comparing evolvability and variability of quantitative traits. Genetics 130:195–204. 1732160 10.1093/genetics/130.1.195 PMC1204793

[CR25] Houle D (1998) How should we explain variation in the genetic variance of traits? Genetica 102–103:241. 9720283

[CR26] Houle D, Hughes KA, Hoffmaster DK, Ihara J, Assimacopoulos S, Canada D et al. (1994) The effects of spontaneous mutation on quantitative traits. I. Variances and covariances of life history traits. Genetics 138:773–785. 7851773 10.1093/genetics/138.3.773 PMC1206226

[CR27] Houle D, Morikawa B, Lynch M (1996) Comparing mutational variabilities. Genetics 143:1467–1483. 8807316 10.1093/genetics/143.3.1467 PMC1207413

[CR28] Johnson-Schlitz D, Fischer JA, Schiffman HJ, Scharenbrock AR, Olufs ZPG, Wassarman DA, et al. (2022) Anesthetic preconditioning of traumatic brain injury is ineffective in a drosophila model of obesity. J Pharmacol Exp Ther 381:229–235. 35347062 10.1124/jpet.121.000997 PMC9190232

[CR29] Kals M, Kunzmann K, Parodi L, Radmanesh F, Wilson L, Izzy S et al. (2022) A genome-wide association study of outcome from traumatic brain injury. eBioMedicine 77:103933. 35301180 10.1016/j.ebiom.2022.103933 PMC8927841

[CR30] Katzenberger RJ, Chtarbanova S, Rimkus SA, Fischer JA, Kaur G, Seppala JM et al. (2015) Death following traumatic brain injury in Drosophila is associated with intestinal barrier dysfunction. eLife 4:e04790. 25742603 10.7554/eLife.04790 PMC4377547

[CR31] Katzenberger RJ, Ganetzky B, Wassarman DA (2023) Lissencephaly-1 mutations enhance traumatic brain injury outcomes in Drosophila. Genetics 223:iyad008. 36683334 10.1093/genetics/iyad008 PMC9991514

[CR32] Katzenberger RJ, Loewen CA, Wassarman DR, Petersen AJ, Ganetzky B, Wassarman DA (2013) A Drosophila model of closed head traumatic brain injury. Proc Natl Acad Sci USA 110:E4152–E4159. 24127584 10.1073/pnas.1316895110 PMC3816429

[CR33] Keightley PD, Hill WG (1988) Quantitative genetic variability maintained by mutation-stabilizing selection balance in finite populations. Genet Res 52:33–43. 3181758 10.1017/s0016672300027282

[CR34] Latter BD, Mulley JC, Reid D, Pascoe L (1995) Reduced genetic load revealed by slow inbreeding in Drosophila melanogaster. Genetics 139:287–297. 7705630 10.1093/genetics/139.1.287 PMC1206325

[CR35] Li S, Vazquez JM, Sudmant PH (2023) The evolution of aging and lifespan. Trends Genet 39:830–843. 37714733 10.1016/j.tig.2023.08.005 PMC11147682

[CR36] Lynch M, Walsh B (1998) Genetics and analysis of quantitative traits. Sinauer, Sunderland, MA.

[CR37] Mackay TFC, Richards S, Stone EA, Barbadilla A, Ayroles JF, Zhu D et al. (2012) The Drosophila melanogaster Genetic Reference Panel. Nature 482:173–178. 22318601 10.1038/nature10811 PMC3683990

[CR38] Mallet MA, Chippindale AK (2011) Inbreeding reveals stronger net selection on Drosophila melanogaster males: implications for mutation load and the fitness of sexual females. Heredity 106:994–1002. 21119701 10.1038/hdy.2010.148 PMC3186252

[CR39] Melde RH, Abraham JM, Ugolini MR, Castle MP, Fjalstad MM, Blumstein DM et al. (2024) Sex-specific viability effects of mutations in Drosophila melanogaster. Evolution 78:1844–1853. 39277542 10.1093/evolut/qpae134 PMC12224230

[CR40] Merritt VC, Maihofer AX, Gasperi M, Chanfreau-Coffinier C, Stein MB, Panizzon MS et al. (2024) Genome-wide association study of traumatic brain injury in U.S. military veterans enrolled in the VA Million Veteran Program. Mol Psychiatry 29:97–111. 37875548 10.1038/s41380-023-02304-8

[CR41] Nivard MJ, Pastink A, Vogel EW (1992) Molecular analysis of mutations induced in the vermilion gene of Drosophila melanogaster by methyl methanesulfonate. Genetics 131:673–682. 1628810 10.1093/genetics/131.3.673 PMC1205038

[CR42] Ponsford J (2013) Factors contributing to outcome following traumatic brain injury. NeuroRehabilitation 32:803–815. 23867406 10.3233/NRE-130904

[CR43] Roozenbeek B, Maas AIR, Menon DK (2013) Changing patterns in the epidemiology of traumatic brain injury. Nat Rev Neurol 9:231–236. 23443846 10.1038/nrneurol.2013.22

[CR44] Rowe L, Houle D (1996) The lek paradox and the capture of genetic variance by condition dependent traits. Proc R Soc Lond Ser B: Biol Sci 263:1415–1421.

[CR45] Sanchez CM, Titus DJ, Wilson NM, Freund JE, Atkins CM (2021) Early life stress exacerbates outcome after traumatic brain injury. J Neurotrauma 38:555–565. 32862765 10.1089/neu.2020.7267 PMC8020564

[CR46] Sharp NP, Agrawal AF (2018) An experimental test of the mutation-selection balance model for the maintenance of genetic variance in fitness components. Proc Royal Soc B 285:20181864. 10.1098/rspb.2018.1864 PMC6235037 30404880

[CR47] Swanson LC, Trujillo EA, Thiede GH, Katzenberger RJ, Shishkova E, Coon JJ et al. (2020) Survival following traumatic brain injury in drosophila is increased by heterozygosity for a mutation of the NF-κB innate immune response transcription factor relish. Genetics 216:1117–1136. 33109529 10.1534/genetics.120.303776 PMC7768241

[CR48] Tomkins JL, Radwan J, Kotiaho JS, Tregenza T (2004) Genic capture and resolving the lek paradox. Trends Ecol Evol 19:323–328. 16701278 10.1016/j.tree.2004.03.029

[CR49] Walsh B, Lynch M (2018) Evolution and selection of quantitative traits. Oxford University Press, Oxford UK.

[CR50] Zhang X-S, Hill WG (2005) Genetic variability under mutation selection balance. Trends Ecol Evol 20:468–470. 16701419 10.1016/j.tree.2005.06.010

